# Supplementary material for: Modeling glioblastoma heterogeneity as a dynamic network of cell states
Source: Mol Syst Biol. 2021 Sep 16;17(9):e10105. doi: 10.15252/msb.202010105 (PMC8444284; doi:10.15252/msb.202010105)
Supplement: Supplementary file 5 — Source Data for Figure 3 [file MSB-17-e10105-s001.zip › Figure3A_sourcedata/GSEA_3065/nosigdb_state2.GseaPreranked.1622718419215/RICHARDS_NOWAKOWSKI_SCIENCE_2017_MGE_RG2_UPREG.html]

Details for gene set RICHARDS\_NOWAKOWSKI\_SCIENCE\_2017\_MGE\_RG2\_UPREG[GSEA]

|  || Dataset | state2 |
| Phenotype | NoPhenotypeAvailable |
| Upregulated in class | na\_pos |
| GeneSet | RICHARDS\_NOWAKOWSKI\_SCIENCE\_2017\_MGE\_RG2\_UPREG |
| Enrichment Score (ES) | 0.6928927 |
| Normalized Enrichment Score (NES) | 2.6643412 |
| Nominal p-value | 0.0 |
| FDR q-value | 0.0 |
| FWER p-Value | 0.0 |
Table: GSEA Results Summary

  

Fig 1: Enrichment plot: RICHARDS\_NOWAKOWSKI\_SCIENCE\_2017\_MGE\_RG2\_UPREG      
 Profile of the Running ES Score & Positions of GeneSet Members on the Rank Ordered List

  

| PROBE | GENE SYMBOL | GENE\_TITLE | RANK IN GENE LIST | RANK METRIC SCORE | RUNNING ES | CORE ENRICHMENT || 1 | HMGB2 |  |  | 4 | 0.823 | 0.0102 | Yes |
| 2 | TYMS |  |  | 5 | 0.792 | 0.0205 | Yes |
| 3 | CENPU |  |  | 6 | 0.763 | 0.0304 | Yes |
| 4 | H2AFZ |  |  | 8 | 0.735 | 0.0398 | Yes |
| 5 | DHFR |  |  | 11 | 0.644 | 0.0479 | Yes |
| 6 | MCM7 |  |  | 13 | 0.626 | 0.0559 | Yes |
| 7 | PCNA |  |  | 16 | 0.595 | 0.0634 | Yes |
| 8 | MKI67 |  |  | 17 | 0.590 | 0.0710 | Yes |
| 9 | CDK1 |  |  | 18 | 0.577 | 0.0785 | Yes |
| 10 | MAD2L1 |  |  | 20 | 0.563 | 0.0857 | Yes |
| 11 | ATAD2 |  |  | 21 | 0.563 | 0.0929 | Yes |
| 12 | CKS1B |  |  | 23 | 0.554 | 0.1000 | Yes |
| 13 | DUT |  |  | 24 | 0.552 | 0.1072 | Yes |
| 14 | HMGB1 |  |  | 25 | 0.549 | 0.1143 | Yes |
| 15 | CLSPN |  |  | 27 | 0.536 | 0.1211 | Yes |
| 16 | CKS2 |  |  | 28 | 0.532 | 0.1280 | Yes |
| 17 | ESCO2 |  |  | 30 | 0.530 | 0.1347 | Yes |
| 18 | DEK |  |  | 31 | 0.526 | 0.1415 | Yes |
| 19 | FANCI |  |  | 32 | 0.521 | 0.1483 | Yes |
| 20 | CENPF |  |  | 34 | 0.518 | 0.1549 | Yes |
| 21 | HMGN2 |  |  | 35 | 0.512 | 0.1615 | Yes |
| 22 | GINS2 |  |  | 37 | 0.506 | 0.1680 | Yes |
| 23 | HIST1H1C |  |  | 38 | 0.498 | 0.1744 | Yes |
| 24 | SMC2 |  |  | 39 | 0.493 | 0.1808 | Yes |
| 25 | ORC6 |  |  | 40 | 0.493 | 0.1872 | Yes |
| 26 | DBF4 |  |  | 41 | 0.492 | 0.1936 | Yes |
| 27 | SMC4 |  |  | 42 | 0.489 | 0.1999 | Yes |
| 28 | TMPO |  |  | 43 | 0.489 | 0.2062 | Yes |
| 29 | CENPK |  |  | 44 | 0.488 | 0.2125 | Yes |
| 30 | NUSAP1 |  |  | 47 | 0.483 | 0.2186 | Yes |
| 31 | PTMA |  |  | 49 | 0.474 | 0.2246 | Yes |
| 32 | MCM3 |  |  | 51 | 0.464 | 0.2305 | Yes |
| 33 | TUBA1B |  |  | 52 | 0.462 | 0.2365 | Yes |
| 34 | RFC2 |  |  | 53 | 0.460 | 0.2424 | Yes |
| 35 | CHAF1A |  |  | 55 | 0.457 | 0.2482 | Yes |
| 36 | UHRF1 |  |  | 57 | 0.454 | 0.2540 | Yes |
| 37 | BIRC5 |  |  | 61 | 0.443 | 0.2594 | Yes |
| 38 | GMNN |  |  | 64 | 0.435 | 0.2648 | Yes |
| 39 | RANBP1 |  |  | 65 | 0.430 | 0.2704 | Yes |
| 40 | RRM1 |  |  | 72 | 0.412 | 0.2751 | Yes |
| 41 | SNRPD1 |  |  | 74 | 0.408 | 0.2802 | Yes |
| 42 | NUCKS1 |  |  | 78 | 0.404 | 0.2851 | Yes |
| 43 | MCM4 |  |  | 79 | 0.399 | 0.2903 | Yes |
| 44 | MCM5 |  |  | 83 | 0.394 | 0.2950 | Yes |
| 45 | KIF15 |  |  | 86 | 0.392 | 0.2999 | Yes |
| 46 | HNRNPD |  |  | 88 | 0.390 | 0.3048 | Yes |
| 47 | RAD51AP1 |  |  | 90 | 0.388 | 0.3097 | Yes |
| 48 | ALYREF |  |  | 91 | 0.382 | 0.3147 | Yes |
| 49 | BRCA1 |  |  | 92 | 0.381 | 0.3196 | Yes |
| 50 | RAN |  |  | 98 | 0.375 | 0.3239 | Yes |
| 51 | TMSB15A |  |  | 99 | 0.374 | 0.3288 | Yes |
| 52 | MCM10 |  |  | 100 | 0.373 | 0.3336 | Yes |
| 53 | FEN1 |  |  | 107 | 0.371 | 0.3377 | Yes |
| 54 | VRK1 |  |  | 118 | 0.350 | 0.3412 | Yes |
| 55 | POLD3 |  |  | 120 | 0.347 | 0.3455 | Yes |
| 56 | DNMT1 |  |  | 121 | 0.345 | 0.3500 | Yes |
| 57 | YBX1 |  |  | 124 | 0.340 | 0.3542 | Yes |
| 58 | RFC4 |  |  | 126 | 0.339 | 0.3585 | Yes |
| 59 | CDK4 |  |  | 131 | 0.333 | 0.3623 | Yes |
| 60 | SNRPB |  |  | 132 | 0.332 | 0.3666 | Yes |
| 61 | ANP32E |  |  | 136 | 0.330 | 0.3706 | Yes |
| 62 | PA2G4 |  |  | 137 | 0.329 | 0.3748 | Yes |
| 63 | USP1 |  |  | 138 | 0.329 | 0.3791 | Yes |
| 64 | SET |  |  | 139 | 0.328 | 0.3834 | Yes |
| 65 | SRSF3 |  |  | 140 | 0.327 | 0.3876 | Yes |
| 66 | RBBP8 |  |  | 141 | 0.325 | 0.3918 | Yes |
| 67 | NCL |  |  | 146 | 0.316 | 0.3954 | Yes |
| 68 | DTL |  |  | 148 | 0.311 | 0.3994 | Yes |
| 69 | MCM6 |  |  | 149 | 0.311 | 0.4034 | Yes |
| 70 | KIF22 |  |  | 154 | 0.306 | 0.4069 | Yes |
| 71 | SRSF7 |  |  | 155 | 0.306 | 0.4109 | Yes |
| 72 | NASP |  |  | 156 | 0.306 | 0.4149 | Yes |
| 73 | HELLS |  |  | 161 | 0.304 | 0.4183 | Yes |
| 74 | TMEM106C |  |  | 162 | 0.303 | 0.4223 | Yes |
| 75 | CHCHD2 |  |  | 165 | 0.302 | 0.4260 | Yes |
| 76 | SLC25A5 |  |  | 168 | 0.299 | 0.4296 | Yes |
| 77 | HSPD1 |  |  | 176 | 0.294 | 0.4326 | Yes |
| 78 | ITGB3BP |  |  | 177 | 0.294 | 0.4364 | Yes |
| 79 | OLIG2 |  |  | 179 | 0.294 | 0.4401 | Yes |
| 80 | SRI |  |  | 181 | 0.293 | 0.4438 | Yes |
| 81 | BARD1 |  |  | 185 | 0.292 | 0.4473 | Yes |
| 82 | PAICS |  |  | 187 | 0.291 | 0.4509 | Yes |
| 83 | PPIA |  |  | 189 | 0.287 | 0.4545 | Yes |
| 84 | FAM111B |  |  | 191 | 0.285 | 0.4581 | Yes |
| 85 | HAUS1 |  |  | 192 | 0.285 | 0.4618 | Yes |
| 86 | PARP1 |  |  | 194 | 0.284 | 0.4654 | Yes |
| 87 | HNRNPF |  |  | 205 | 0.279 | 0.4679 | Yes |
| 88 | NME1 |  |  | 214 | 0.274 | 0.4705 | Yes |
| 89 | C19orf48 |  |  | 215 | 0.274 | 0.4741 | Yes |
| 90 | LSM2 |  |  | 219 | 0.272 | 0.4773 | Yes |
| 91 | CHEK1 |  |  | 224 | 0.266 | 0.4803 | Yes |
| 92 | CBX5 |  |  | 225 | 0.265 | 0.4837 | Yes |
| 93 | CCND2 |  |  | 227 | 0.263 | 0.4870 | Yes |
| 94 | MCM2 |  |  | 228 | 0.262 | 0.4904 | Yes |
| 95 | HNRNPUL1 |  |  | 248 | 0.253 | 0.4916 | Yes |
| 96 | SMC1A |  |  | 249 | 0.253 | 0.4948 | Yes |
| 97 | MIS18BP1 |  |  | 257 | 0.246 | 0.4972 | Yes |
| 98 | LIG1 |  |  | 259 | 0.246 | 0.5003 | Yes |
| 99 | RAD23A |  |  | 264 | 0.245 | 0.5031 | Yes |
| 100 | PHGDH |  |  | 271 | 0.243 | 0.5055 | Yes |
| 101 | TIMELESS |  |  | 272 | 0.243 | 0.5087 | Yes |
| 102 | CHCHD3 |  |  | 277 | 0.242 | 0.5114 | Yes |
| 103 | CCT5 |  |  | 279 | 0.242 | 0.5144 | Yes |
| 104 | ANAPC11 |  |  | 282 | 0.240 | 0.5173 | Yes |
| 105 | CCNE2 |  |  | 288 | 0.236 | 0.5198 | Yes |
| 106 | NCAPD3 |  |  | 294 | 0.234 | 0.5223 | Yes |
| 107 | NME4 |  |  | 295 | 0.234 | 0.5253 | Yes |
| 108 | RFC5 |  |  | 297 | 0.233 | 0.5282 | Yes |
| 109 | EXOSC9 |  |  | 298 | 0.233 | 0.5312 | Yes |
| 110 | TPI1 |  |  | 301 | 0.231 | 0.5340 | Yes |
| 111 | GSTP1 |  |  | 306 | 0.230 | 0.5365 | Yes |
| 112 | CEBPZ |  |  | 310 | 0.229 | 0.5391 | Yes |
| 113 | RPL41 |  |  | 313 | 0.227 | 0.5419 | Yes |
| 114 | RAD18 |  |  | 317 | 0.227 | 0.5445 | Yes |
| 115 | HNRNPA3 |  |  | 320 | 0.226 | 0.5472 | Yes |
| 116 | TMEM97 |  |  | 331 | 0.223 | 0.5489 | Yes |
| 117 | LDHB |  |  | 343 | 0.219 | 0.5506 | Yes |
| 118 | NPM1 |  |  | 351 | 0.216 | 0.5526 | Yes |
| 119 | BLM |  |  | 355 | 0.215 | 0.5550 | Yes |
| 120 | GLO1 |  |  | 363 | 0.214 | 0.5570 | Yes |
| 121 | RPA2 |  |  | 367 | 0.212 | 0.5594 | Yes |
| 122 | H2AFY |  |  | 376 | 0.209 | 0.5613 | Yes |
| 123 | MSH6 |  |  | 380 | 0.209 | 0.5636 | Yes |
| 124 | HAT1 |  |  | 381 | 0.209 | 0.5663 | Yes |
| 125 | CDK2 |  |  | 382 | 0.208 | 0.5690 | Yes |
| 126 | NAP1L1 |  |  | 387 | 0.207 | 0.5713 | Yes |
| 127 | GNG5 |  |  | 389 | 0.207 | 0.5738 | Yes |
| 128 | NOP56 |  |  | 396 | 0.201 | 0.5758 | Yes |
| 129 | NUTF2 |  |  | 397 | 0.201 | 0.5784 | Yes |
| 130 | UNG |  |  | 398 | 0.200 | 0.5810 | Yes |
| 131 | RPL23A |  |  | 406 | 0.199 | 0.5828 | Yes |
| 132 | RPS25 |  |  | 413 | 0.197 | 0.5847 | Yes |
| 133 | RPL35 |  |  | 415 | 0.197 | 0.5871 | Yes |
| 134 | BRIP1 |  |  | 426 | 0.194 | 0.5885 | Yes |
| 135 | BRCA2 |  |  | 427 | 0.193 | 0.5910 | Yes |
| 136 | RHOA |  |  | 442 | 0.192 | 0.5919 | Yes |
| 137 | JUND |  |  | 443 | 0.192 | 0.5944 | Yes |
| 138 | FANCD2 |  |  | 447 | 0.191 | 0.5965 | Yes |
| 139 | FAM111A |  |  | 448 | 0.191 | 0.5990 | Yes |
| 140 | MZT2B |  |  | 450 | 0.190 | 0.6014 | Yes |
| 141 | RPLP0 |  |  | 451 | 0.190 | 0.6038 | Yes |
| 142 | EXOSC8 |  |  | 456 | 0.189 | 0.6058 | Yes |
| 143 | DERA |  |  | 471 | 0.185 | 0.6067 | Yes |
| 144 | SSB |  |  | 478 | 0.184 | 0.6084 | Yes |
| 145 | AIF1L |  |  | 481 | 0.183 | 0.6105 | Yes |
| 146 | RSL1D1 |  |  | 484 | 0.183 | 0.6127 | Yes |
| 147 | RPS15 |  |  | 492 | 0.182 | 0.6143 | Yes |
| 148 | LDHA |  |  | 506 | 0.179 | 0.6151 | Yes |
| 149 | HNRNPA2B1 |  |  | 510 | 0.178 | 0.6171 | Yes |
| 150 | PRC1 |  |  | 514 | 0.178 | 0.6191 | Yes |
| 151 | LRR1 |  |  | 516 | 0.178 | 0.6213 | Yes |
| 152 | RPL7 |  |  | 518 | 0.178 | 0.6235 | Yes |
| 153 | RPL18A |  |  | 523 | 0.177 | 0.6253 | Yes |
| 154 | RPSA |  |  | 533 | 0.176 | 0.6266 | Yes |
| 155 | UQCRH |  |  | 535 | 0.175 | 0.6288 | Yes |
| 156 | SNRPD2 |  |  | 551 | 0.172 | 0.6293 | Yes |
| 157 | RPL8 |  |  | 553 | 0.172 | 0.6314 | Yes |
| 158 | RPS8 |  |  | 584 | 0.166 | 0.6302 | Yes |
| 159 | DKC1 |  |  | 592 | 0.165 | 0.6316 | Yes |
| 160 | RPS3 |  |  | 598 | 0.164 | 0.6332 | Yes |
| 161 | CNBP |  |  | 599 | 0.164 | 0.6353 | Yes |
| 162 | RPL12 |  |  | 611 | 0.162 | 0.6361 | Yes |
| 163 | RFC3 |  |  | 617 | 0.161 | 0.6377 | Yes |
| 164 | RPS16 |  |  | 625 | 0.159 | 0.6390 | Yes |
| 165 | TIMM10 |  |  | 639 | 0.158 | 0.6395 | Yes |
| 166 | CKB |  |  | 647 | 0.157 | 0.6408 | Yes |
| 167 | RPLP1 |  |  | 648 | 0.156 | 0.6428 | Yes |
| 168 | RFC1 |  |  | 655 | 0.156 | 0.6442 | Yes |
| 169 | PRIM1 |  |  | 660 | 0.155 | 0.6457 | Yes |
| 170 | RPL32 |  |  | 661 | 0.155 | 0.6477 | Yes |
| 171 | NUDT5 |  |  | 663 | 0.155 | 0.6496 | Yes |
| 172 | SRSF10 |  |  | 664 | 0.155 | 0.6516 | Yes |
| 173 | RPL29 |  |  | 673 | 0.153 | 0.6527 | Yes |
| 174 | SNRPC |  |  | 680 | 0.152 | 0.6540 | Yes |
| 175 | LSM6 |  |  | 697 | 0.151 | 0.6542 | Yes |
| 176 | WDR76 |  |  | 702 | 0.150 | 0.6557 | Yes |
| 177 | ENO1 |  |  | 717 | 0.148 | 0.6561 | Yes |
| 178 | RPS3A |  |  | 719 | 0.148 | 0.6579 | Yes |
| 179 | E2F7 |  |  | 721 | 0.148 | 0.6597 | Yes |
| 180 | FBL |  |  | 738 | 0.146 | 0.6598 | Yes |
| 181 | POLA1 |  |  | 752 | 0.143 | 0.6602 | Yes |
| 182 | SRRM1 |  |  | 757 | 0.143 | 0.6616 | Yes |
| 183 | RPS13 |  |  | 761 | 0.143 | 0.6631 | Yes |
| 184 | MNS1 |  |  | 774 | 0.142 | 0.6636 | Yes |
| 185 | EEF1D |  |  | 783 | 0.140 | 0.6645 | Yes |
| 186 | MMS22L |  |  | 799 | 0.138 | 0.6647 | Yes |
| 187 | PRTFDC1 |  |  | 818 | 0.137 | 0.6644 | Yes |
| 188 | RPS6 |  |  | 819 | 0.136 | 0.6662 | Yes |
| 189 | EMP2 |  |  | 826 | 0.135 | 0.6673 | Yes |
| 190 | RPS14 |  |  | 834 | 0.134 | 0.6682 | Yes |
| 191 | HNRNPC |  |  | 850 | 0.133 | 0.6683 | Yes |
| 192 | RPL27 |  |  | 852 | 0.132 | 0.6699 | Yes |
| 193 | SEPT10 |  |  | 869 | 0.131 | 0.6698 | Yes |
| 194 | HNRNPA1 |  |  | 876 | 0.130 | 0.6708 | Yes |
| 195 | PRDX3 |  |  | 877 | 0.130 | 0.6725 | Yes |
| 196 | MMAB |  |  | 911 | 0.127 | 0.6705 | Yes |
| 197 | GAPDH |  |  | 933 | 0.125 | 0.6698 | Yes |
| 198 | RPL15 |  |  | 936 | 0.125 | 0.6712 | Yes |
| 199 | NUP62 |  |  | 957 | 0.123 | 0.6705 | Yes |
| 200 | RPL13 |  |  | 958 | 0.123 | 0.6721 | Yes |
| 201 | RPL6 |  |  | 968 | 0.122 | 0.6727 | Yes |
| 202 | COA4 |  |  | 976 | 0.122 | 0.6735 | Yes |
| 203 | RPL10A |  |  | 984 | 0.121 | 0.6743 | Yes |
| 204 | PLK4 |  |  | 986 | 0.121 | 0.6758 | Yes |
| 205 | PRDX2 |  |  | 990 | 0.121 | 0.6770 | Yes |
| 206 | RPL31 |  |  | 1000 | 0.120 | 0.6775 | Yes |
| 207 | BTF3 |  |  | 1017 | 0.118 | 0.6773 | Yes |
| 208 | GPATCH4 |  |  | 1031 | 0.117 | 0.6774 | Yes |
| 209 | SOX2 |  |  | 1036 | 0.117 | 0.6784 | Yes |
| 210 | NEDD1 |  |  | 1039 | 0.116 | 0.6797 | Yes |
| 211 | CEP152 |  |  | 1047 | 0.116 | 0.6804 | Yes |
| 212 | EIF2S1 |  |  | 1051 | 0.115 | 0.6816 | Yes |
| 213 | HAUS6 |  |  | 1054 | 0.115 | 0.6828 | Yes |
| 214 | RPS27A |  |  | 1072 | 0.113 | 0.6824 | Yes |
| 215 | RPL7A |  |  | 1073 | 0.113 | 0.6839 | Yes |
| 216 | RPL39 |  |  | 1074 | 0.113 | 0.6853 | Yes |
| 217 | CCDC138 |  |  | 1081 | 0.112 | 0.6861 | Yes |
| 218 | GNL3 |  |  | 1084 | 0.112 | 0.6874 | Yes |
| 219 | RPL27A |  |  | 1085 | 0.112 | 0.6888 | Yes |
| 220 | WDHD1 |  |  | 1092 | 0.112 | 0.6896 | Yes |
| 221 | RPLP2 |  |  | 1099 | 0.112 | 0.6904 | Yes |
| 222 | RPL21 |  |  | 1123 | 0.110 | 0.6893 | Yes |
| 223 | APBB2 |  |  | 1130 | 0.110 | 0.6900 | Yes |
| 224 | CCDC59 |  |  | 1165 | 0.107 | 0.6876 | Yes |
| 225 | RPL9 |  |  | 1166 | 0.107 | 0.6890 | Yes |
| 226 | SRRM2 |  |  | 1178 | 0.106 | 0.6891 | Yes |
| 227 | EIF3M |  |  | 1182 | 0.106 | 0.6902 | Yes |
| 228 | STAG2 |  |  | 1198 | 0.105 | 0.6899 | Yes |
| 229 | RPL4 |  |  | 1199 | 0.105 | 0.6912 | Yes |
| 230 | RDX |  |  | 1214 | 0.104 | 0.6910 | Yes |
| 231 | PRPS2 |  |  | 1221 | 0.103 | 0.6917 | Yes |
| 232 | CTPS1 |  |  | 1223 | 0.103 | 0.6929 | Yes |
| 233 | DDAH1 |  |  | 1260 | 0.101 | 0.6902 | No |
| 234 | RPL13A |  |  | 1279 | 0.100 | 0.6895 | No |
| 235 | CLIC1 |  |  | 1291 | 0.099 | 0.6895 | No |
| 236 | FDPS |  |  | 1297 | 0.098 | 0.6903 | No |
| 237 | HES6 |  |  | 1305 | 0.098 | 0.6907 | No |
| 238 | SRSF1 |  |  | 1315 | 0.097 | 0.6910 | No |
| 239 | CDC5L |  |  | 1337 | 0.096 | 0.6899 | No |
| 240 | RPL23 |  |  | 1339 | 0.096 | 0.6910 | No |
| 241 | SKP2 |  |  | 1362 | 0.094 | 0.6898 | No |
| 242 | CNTLN |  |  | 1386 | 0.093 | 0.6884 | No |
| 243 | POLE2 |  |  | 1388 | 0.093 | 0.6895 | No |
| 244 | CEP83 |  |  | 1405 | 0.092 | 0.6890 | No |
| 245 | DAXX |  |  | 1425 | 0.091 | 0.6880 | No |
| 246 | RPL36 |  |  | 1430 | 0.091 | 0.6887 | No |
| 247 | CCT3 |  |  | 1432 | 0.090 | 0.6898 | No |
| 248 | LIN54 |  |  | 1440 | 0.090 | 0.6902 | No |
| 249 | SOX9 |  |  | 1466 | 0.088 | 0.6886 | No |
| 250 | NACA |  |  | 1482 | 0.087 | 0.6880 | No |
| 251 | FAM161A |  |  | 1523 | 0.085 | 0.6847 | No |
| 252 | RPL18 |  |  | 1526 | 0.085 | 0.6856 | No |
| 253 | G3BP1 |  |  | 1566 | 0.083 | 0.6823 | No |
| 254 | RBL1 |  |  | 1569 | 0.083 | 0.6832 | No |
| 255 | C12orf29 |  |  | 1572 | 0.083 | 0.6840 | No |
| 256 | PABPC1 |  |  | 1589 | 0.082 | 0.6833 | No |
| 257 | RPL3 |  |  | 1590 | 0.082 | 0.6843 | No |
| 258 | WEE1 |  |  | 1617 | 0.081 | 0.6825 | No |
| 259 | RPS4X |  |  | 1631 | 0.080 | 0.6821 | No |
| 260 | YBX3 |  |  | 1677 | 0.078 | 0.6781 | No |
| 261 | RPS27 |  |  | 1683 | 0.078 | 0.6786 | No |
| 262 | CDCA7 |  |  | 1684 | 0.078 | 0.6796 | No |
| 263 | LITAF |  |  | 1685 | 0.078 | 0.6806 | No |
| 264 | EGR1 |  |  | 1691 | 0.077 | 0.6810 | No |
| 265 | MRPL24 |  |  | 1700 | 0.077 | 0.6811 | No |
| 266 | RPS28 |  |  | 1743 | 0.075 | 0.6774 | No |
| 267 | SF3A3 |  |  | 1767 | 0.074 | 0.6758 | No |
| 268 | SNX4 |  |  | 1782 | 0.073 | 0.6752 | No |
| 269 | PDLIM3 |  |  | 1841 | 0.071 | 0.6697 | No |
| 270 | RPL5 |  |  | 1842 | 0.071 | 0.6706 | No |
| 271 | CDC7 |  |  | 1846 | 0.070 | 0.6712 | No |
| 272 | NAA50 |  |  | 1855 | 0.070 | 0.6712 | No |
| 273 | LYRM2 |  |  | 1859 | 0.070 | 0.6718 | No |
| 274 | MIS12 |  |  | 1864 | 0.070 | 0.6722 | No |
| 275 | PBX3 |  |  | 1923 | 0.067 | 0.6667 | No |
| 276 | RPS18 |  |  | 1926 | 0.067 | 0.6673 | No |
| 277 | CENPC |  |  | 1932 | 0.067 | 0.6676 | No |
| 278 | RPS10 |  |  | 1973 | 0.066 | 0.6640 | No |
| 279 | SERF2 |  |  | 1979 | 0.065 | 0.6643 | No |
| 280 | ZEB1 |  |  | 1995 | 0.065 | 0.6635 | No |
| 281 | NUP153 |  |  | 2025 | 0.064 | 0.6611 | No |
| 282 | RPL37A |  |  | 2075 | 0.062 | 0.6564 | No |
| 283 | CDK6 |  |  | 2124 | 0.060 | 0.6519 | No |
| 284 | PRKDC |  |  | 2154 | 0.059 | 0.6494 | No |
| 285 | SMAD5 |  |  | 2156 | 0.059 | 0.6501 | No |
| 286 | PEA15 |  |  | 2161 | 0.059 | 0.6504 | No |
| 287 | BAZ1A |  |  | 2193 | 0.058 | 0.6477 | No |
| 288 | APEX1 |  |  | 2239 | 0.056 | 0.6434 | No |
| 289 | PRMT5 |  |  | 2240 | 0.056 | 0.6441 | No |
| 290 | RPS19 |  |  | 2268 | 0.055 | 0.6418 | No |
| 291 | CRKL |  |  | 2285 | 0.054 | 0.6408 | No |
| 292 | ASCL1 |  |  | 2318 | 0.053 | 0.6379 | No |
| 293 | ZFP36L1 |  |  | 2320 | 0.053 | 0.6385 | No |
| 294 | DPH3 |  |  | 2343 | 0.052 | 0.6367 | No |
| 295 | SALL1 |  |  | 2372 | 0.051 | 0.6343 | No |
| 296 | TMA16 |  |  | 2393 | 0.051 | 0.6327 | No |
| 297 | G2E3 |  |  | 2405 | 0.051 | 0.6321 | No |
| 298 | KNTC1 |  |  | 2440 | 0.050 | 0.6290 | No |
| 299 | ZRANB3 |  |  | 2450 | 0.049 | 0.6286 | No |
| 300 | ARL4A |  |  | 2510 | 0.047 | 0.6227 | No |
| 301 | ANXA7 |  |  | 2542 | 0.046 | 0.6198 | No |
| 302 | GLTP |  |  | 2553 | 0.046 | 0.6193 | No |
| 303 | HAUS5 |  |  | 2575 | 0.045 | 0.6176 | No |
| 304 | HSDL2 |  |  | 2644 | 0.043 | 0.6106 | No |
| 305 | ING5 |  |  | 2725 | 0.041 | 0.6022 | No |
| 306 | RPL10 |  |  | 2755 | 0.040 | 0.5995 | No |
| 307 | ANXA5 |  |  | 2812 | 0.039 | 0.5938 | No |
| 308 | STK39 |  |  | 2834 | 0.038 | 0.5919 | No |
| 309 | C1orf35 |  |  | 2852 | 0.038 | 0.5905 | No |
| 310 | ALDH6A1 |  |  | 2912 | 0.036 | 0.5844 | No |
| 311 | ZNF718 |  |  | 3092 | 0.032 | 0.5649 | No |
| 312 | BMS1 |  |  | 3100 | 0.032 | 0.5646 | No |
| 313 | ZFP36L2 |  |  | 3122 | 0.031 | 0.5626 | No |
| 314 | NSRP1 |  |  | 3175 | 0.030 | 0.5573 | No |
| 315 | ARHGAP5 |  |  | 3234 | 0.029 | 0.5512 | No |
| 316 | PPIL4 |  |  | 3424 | 0.025 | 0.5305 | No |
| 317 | WRN |  |  | 3435 | 0.025 | 0.5297 | No |
| 318 | NR1D2 |  |  | 3471 | 0.024 | 0.5261 | No |
| 319 | TP53 |  |  | 3495 | 0.024 | 0.5239 | No |
| 320 | ALDH1B1 |  |  | 3502 | 0.024 | 0.5235 | No |
| 321 | TROVE2 |  |  | 3514 | 0.024 | 0.5226 | No |
| 322 | HSPB1 |  |  | 3629 | 0.022 | 0.5102 | No |
| 323 | PNN |  |  | 3632 | 0.022 | 0.5103 | No |
| 324 | TLK1 |  |  | 3656 | 0.021 | 0.5080 | No |
| 325 | DLEU2 |  |  | 3706 | 0.020 | 0.5028 | No |
| 326 | PARD3 |  |  | 3728 | 0.020 | 0.5007 | No |
| 327 | DCAF16 |  |  | 3875 | 0.018 | 0.4847 | No |
| 328 | NUP43 |  |  | 4017 | 0.015 | 0.4693 | No |
| 329 | MBIP |  |  | 4066 | 0.014 | 0.4641 | No |
| 330 | C5orf30 |  |  | 4107 | 0.014 | 0.4599 | No |
| 331 | SALL3 |  |  | 4113 | 0.014 | 0.4595 | No |
| 332 | YAP1 |  |  | 4184 | 0.012 | 0.4519 | No |
| 333 | BAG4 |  |  | 4276 | 0.011 | 0.4419 | No |
| 334 | MRPL45 |  |  | 4292 | 0.010 | 0.4403 | No |
| 335 | TMEM18 |  |  | 4476 | 0.008 | 0.4201 | No |
| 336 | GLCCI1 |  |  | 4492 | 0.007 | 0.4185 | No |
| 337 | CDO1 |  |  | 4513 | 0.007 | 0.4164 | No |
| 338 | SRSF6 |  |  | 4555 | 0.006 | 0.4119 | No |
| 339 | IQGAP2 |  |  | 4559 | 0.006 | 0.4117 | No |
| 340 | UTP20 |  |  | 4703 | 0.004 | 0.3958 | No |
| 341 | DGKH |  |  | 4858 | 0.002 | 0.3787 | No |
| 342 | PKM |  |  | 4880 | 0.001 | 0.3764 | No |
| 343 | LARP1B |  |  | 4972 | 0.000 | 0.3663 | No |
| 344 | PHF10 |  |  | 5073 | -0.001 | 0.3552 | No |
| 345 | DIS3L |  |  | 5087 | -0.001 | 0.3538 | No |
| 346 | EIF3E |  |  | 5166 | -0.003 | 0.3451 | No |
| 347 | MTA3 |  |  | 5218 | -0.004 | 0.3395 | No |
| 348 | CHIC2 |  |  | 5329 | -0.005 | 0.3274 | No |
| 349 | CDC37 |  |  | 5466 | -0.008 | 0.3123 | No |
| 350 | FAM114A1 |  |  | 5495 | -0.008 | 0.3093 | No |
| 351 | INPP5F |  |  | 5513 | -0.009 | 0.3076 | No |
| 352 | TRIM9 |  |  | 5580 | -0.010 | 0.3003 | No |
| 353 | CCDC14 |  |  | 5679 | -0.011 | 0.2896 | No |
| 354 | TRIM26 |  |  | 5716 | -0.012 | 0.2857 | No |
| 355 | GAB1 |  |  | 5722 | -0.012 | 0.2853 | No |
| 356 | TRMT10B |  |  | 5781 | -0.013 | 0.2791 | No |
| 357 | STARD9 |  |  | 5784 | -0.013 | 0.2790 | No |
| 358 | ZNF121 |  |  | 5820 | -0.013 | 0.2753 | No |
| 359 | TOX3 |  |  | 5867 | -0.014 | 0.2704 | No |
| 360 | CHD7 |  |  | 5923 | -0.015 | 0.2644 | No |
| 361 | PTPN13 |  |  | 5985 | -0.016 | 0.2579 | No |
| 362 | PIKFYVE |  |  | 6052 | -0.017 | 0.2508 | No |
| 363 | AHI1 |  |  | 6071 | -0.018 | 0.2490 | No |
| 364 | SCAF11 |  |  | 6095 | -0.018 | 0.2467 | No |
| 365 | TCF7L1 |  |  | 6384 | -0.023 | 0.2150 | No |
| 366 | PDPN |  |  | 6446 | -0.024 | 0.2085 | No |
| 367 | NES |  |  | 6455 | -0.025 | 0.2079 | No |
| 368 | ILDR2 |  |  | 6458 | -0.025 | 0.2080 | No |
| 369 | ABAT |  |  | 6574 | -0.027 | 0.1956 | No |
| 370 | FOXN3 |  |  | 6582 | -0.027 | 0.1952 | No |
| 371 | EEF1A1 |  |  | 6690 | -0.029 | 0.1836 | No |
| 372 | ALG8 |  |  | 6721 | -0.030 | 0.1807 | No |
| 373 | MDK |  |  | 6783 | -0.031 | 0.1743 | No |
| 374 | TMX1 |  |  | 6847 | -0.033 | 0.1677 | No |
| 375 | SLC35F1 |  |  | 6914 | -0.035 | 0.1608 | No |
| 376 | JAM2 |  |  | 6939 | -0.035 | 0.1586 | No |
| 377 | POLI |  |  | 6965 | -0.035 | 0.1563 | No |
| 378 | FUT8 |  |  | 7194 | -0.042 | 0.1315 | No |
| 379 | NOTCH3 |  |  | 7196 | -0.042 | 0.1319 | No |
| 380 | BBX |  |  | 7215 | -0.042 | 0.1305 | No |
| 381 | FOS |  |  | 7222 | -0.043 | 0.1304 | No |
| 382 | ZC3HAV1 |  |  | 7233 | -0.043 | 0.1298 | No |
| 383 | TMEM263 |  |  | 7263 | -0.044 | 0.1272 | No |
| 384 | PCDH17 |  |  | 7304 | -0.045 | 0.1233 | No |
| 385 | IGSF11 |  |  | 7343 | -0.046 | 0.1197 | No |
| 386 | PTPRN2 |  |  | 7358 | -0.046 | 0.1187 | No |
| 387 | PSD3 |  |  | 7470 | -0.050 | 0.1070 | No |
| 388 | QKI |  |  | 7478 | -0.050 | 0.1069 | No |
| 389 | LIMD1 |  |  | 7484 | -0.050 | 0.1070 | No |
| 390 | MTCL1 |  |  | 7487 | -0.050 | 0.1074 | No |
| 391 | CTNNB1 |  |  | 7580 | -0.053 | 0.0978 | No |
| 392 | CUTA |  |  | 7582 | -0.053 | 0.0984 | No |
| 393 | PDLIM5 |  |  | 7663 | -0.055 | 0.0902 | No |
| 394 | TLE3 |  |  | 7678 | -0.056 | 0.0894 | No |
| 395 | ZFAND6 |  |  | 7733 | -0.057 | 0.0841 | No |
| 396 | SOAT1 |  |  | 7758 | -0.058 | 0.0822 | No |
| 397 | TMEM123 |  |  | 7833 | -0.061 | 0.0748 | No |
| 398 | SFRP2 |  |  | 7843 | -0.061 | 0.0746 | No |
| 399 | DYNC2H1 |  |  | 7929 | -0.064 | 0.0659 | No |
| 400 | CORO1C |  |  | 7939 | -0.064 | 0.0658 | No |
| 401 | TFDP2 |  |  | 7977 | -0.066 | 0.0625 | No |
| 402 | TSPAN6 |  |  | 8000 | -0.067 | 0.0609 | No |
| 403 | TPM2 |  |  | 8019 | -0.068 | 0.0598 | No |
| 404 | EGFR |  |  | 8094 | -0.071 | 0.0525 | No |
| 405 | SFRP1 |  |  | 8103 | -0.071 | 0.0526 | No |
| 406 | TRIM16 |  |  | 8146 | -0.074 | 0.0488 | No |
| 407 | AASS |  |  | 8209 | -0.078 | 0.0430 | No |
| 408 | CREB5 |  |  | 8279 | -0.082 | 0.0363 | No |
| 409 | GLUL |  |  | 8307 | -0.083 | 0.0344 | No |
| 410 | SPECC1 |  |  | 8346 | -0.085 | 0.0313 | No |
| 411 | FRMD5 |  |  | 8379 | -0.088 | 0.0289 | No |
| 412 | TMSB4X |  |  | 8392 | -0.089 | 0.0287 | No |
| 413 | RFTN2 |  |  | 8418 | -0.090 | 0.0271 | No |
| 414 | MSI2 |  |  | 8667 | -0.108 | 0.0009 | No |
| 415 | WWTR1 |  |  | 8752 | -0.117 | -0.0069 | No |
| 416 | NOTCH1 |  |  | 8763 | -0.118 | -0.0065 | No |
| 417 | PABPN1 |  |  | 8818 | -0.125 | -0.0109 | No |
| 418 | HDAC9 |  |  | 8833 | -0.127 | -0.0108 | No |
| 419 | CYR61 |  |  | 8901 | -0.136 | -0.0165 | No |
| 420 | RCN1 |  |  | 8929 | -0.138 | -0.0177 | No |
| 421 | RIC3 |  |  | 8937 | -0.139 | -0.0167 | No |
| 422 | C9orf3 |  |  | 8953 | -0.142 | -0.0165 | No |
| 423 | SLC16A1 |  |  | 8970 | -0.145 | -0.0164 | No |
| 424 | SCD |  |  | 8982 | -0.146 | -0.0157 | No |
| 425 | AXL |  |  | 9023 | -0.152 | -0.0182 | No |
| 426 | PON2 |  |  | 9030 | -0.154 | -0.0169 | No |
| 427 | LIPG |  |  | 9042 | -0.156 | -0.0161 | No |
| 428 | LDLR |  |  | 9117 | -0.170 | -0.0221 | No |
| 429 | PRDX4 |  |  | 9151 | -0.178 | -0.0235 | No |
| 430 | PDIA6 |  |  | 9165 | -0.180 | -0.0226 | No |
| 431 | FAT1 |  |  | 9207 | -0.193 | -0.0246 | No |
| 432 | FKBP10 |  |  | 9215 | -0.199 | -0.0228 | No |
| 433 | CD99 |  |  | 9224 | -0.203 | -0.0211 | No |
| 434 | PAM |  |  | 9280 | -0.224 | -0.0243 | No |
| 435 | PDIA3 |  |  | 9297 | -0.230 | -0.0231 | No |
| 436 | ZKSCAN1 |  |  | 9306 | -0.233 | -0.0210 | No |
| 437 | SEMA5A |  |  | 9315 | -0.243 | -0.0187 | No |
| 438 | BSG |  |  | 9331 | -0.253 | -0.0171 | No |
| 439 | HSP90B1 |  |  | 9334 | -0.257 | -0.0140 | No |
| 440 | ZBTB20 |  |  | 9341 | -0.261 | -0.0113 | No |
| 441 | ATP1B3 |  |  | 9352 | -0.274 | -0.0089 | No |
| 442 | BCAN |  |  | 9353 | -0.276 | -0.0053 | No |
| 443 | CD63 |  |  | 9394 | -0.353 | -0.0052 | No |
| 444 | TNC |  |  | 9403 | -0.383 | -0.0011 | No |
| 445 | HSPA5 |  |  | 9406 | -0.395 | 0.0038 | No |
Table: GSEA details [plain text format]

  

Fig 2: RICHARDS\_NOWAKOWSKI\_SCIENCE\_2017\_MGE\_RG2\_UPREG: Random ES distribution      
 Gene set null distribution of ES for **RICHARDS\_NOWAKOWSKI\_SCIENCE\_2017\_MGE\_RG2\_UPREG**
